# When is a vesicle not just a vesicle: mitochondrial spheroids and mitochondrial autophagosomes

**DOI:** 10.1186/2045-3701-4-66

**Published:** 2014-11-14

**Authors:** Katherine L Cook, David R Soto-Pantoja, Lu Jin, Mones Abu-Asab, Robert Clarke

**Affiliations:** Department of Oncology and Lombardi Comprehensive Cancer Center, Georgetown University Medical Center, Washington, DC 20057 USA; Laboratory of Pathology, National Cancer Institute, National Institutes of Health, Bethesda, MD 20892 USA; National Eye Institute, National Institutes of Health, Bethesda, MD 20892 USA

## To the Editor

The commentary by Ding and Eskelinen “Do mitochondria donate membrane to form autophagosomes or undergo remodeling to form mitochondrial spheroids?” on our recently published manuscript raises several important points that we wish to address. To do so, we here include several key experiments to clarify further that the mitochondrial vesicles observed in Cook *et al*. are likely to be autophagosomes [1], rather than “mitochondrial spheroids”.

Ding *et al*. previously showed that when mouse embryonic fibroblasts are treated with the mitochondrial de-coupler agent CCCP, a structure they called a “mitochondrial spheroid” develops. These authors concluded that the spheroids are not autophagosomes because they still form in ATG5^−/−^ and ATG7^−/−^ embryonic fibroblasts [2]. However, autophagy is a complex pathway involving multiple mechanisms of activation. While ATG5 and ATG7 can play an important role in autophagosome formation, they are not obligatory in all cases. For example, autophagy can be activated in an ATG5/ATG7 independent manner involving ULK1 and Rab9 [3].

**Figure 1:**
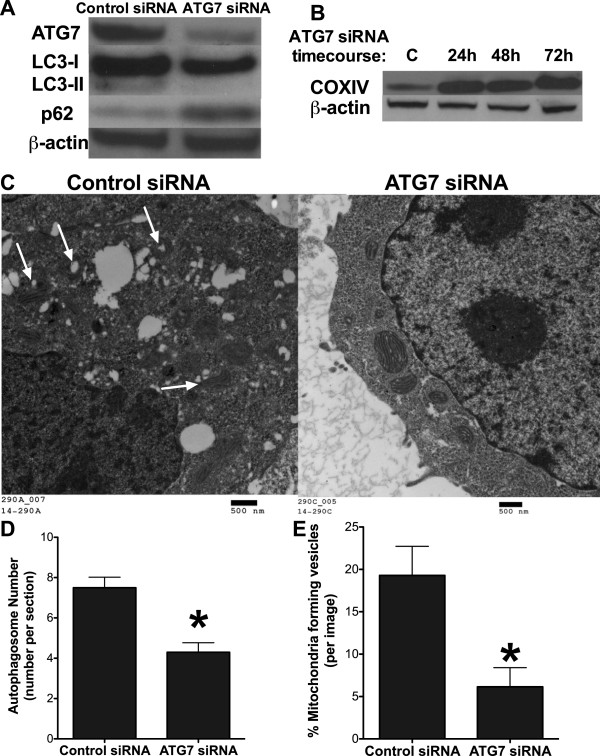
**Effect of autophagy inhibition on mitochondrial vesicle formation. A**. ATG7 knockdown by RNAi was confirmed by Western blot hybridization and ATG7 knockdown inhibited autophagy as determined by LC3-II and p62 protein levels. **B**. Knockdown of ATG7 in LCC9 cells results in accumulation of mitochondria as determined by COXIV protein levels. **C**. EM micrographs of LCC9 cells treated with control or ATG7 siRNA. Arrows denotes mitochondrial forming vesicles. **D**. Autophagosomes were counted from EM images of LCC9 cells treated with control or ATG7 siRNA. n = 10; *p > 0.05. **E**. Mitochondria forming vesicles were counted from EM images of LCC9 cells treated with control or ATG7 siRNA. Data was graphed as % mitochondrial forming vesicles per image. n = 10; *p > 0.05.

Ding and Eskelinen’s criticism of our study is the lack of electron microscopy (EM) images from cells with inhibited autophagy. We now show EM images from LCC9 cells transfected with ATG7 siRNA (Figure 1). We confirmed that ATG7 knockdown inhibits autophagy as shown by a reduction of LC3-II formation and an accumulation of p62 (Figure 1A). Furthermore, EM images indicate that ATG7 knockdown reduces autophagosome formation approximately by 50% (Figure 1D). Knockdown of ATG7 by RNAi also resulted in the accumulation of mitochondria as measured by COXIV (Figure 1B). We also observed increased mitochondria number (average 13.8 mitochondria per EM image versus 9.1 mitochondria per EM image) in ATG7 siRNA transfected cells when compared with control transfected cells. Taken together, these data imply that autophagy is a major pathway for the recycling of mitochondria in antiestrogen resistant breast cancer cells. Moreover, inhibiting autophagy reduced the formation of mitochondrial vesicles, providing further evidence that the vesicles formed by the mitochondria membranes are likely to be autophagosomes (Figure 1C and 1E).

In our previous publication, we demonstrated by immuno-gold electron microscopy, that mitochondria form vesicles that stain positive for LC3, suggesting that these vesicles are likely to be autophagosomes [1]. Microtubule associated protein 1 light chain 3 (MAPLC3, LC3) is lipidated and incorporated into the autophagosomal membrane and is often used as a means to identify appropriate structures as autophagosomes and not lysosomes [4]. ATG7 can play a critical role in LC3 processing and autophagosome formation. Coupled with our new data included here, showing that ATG7 inhibition prevented mitochondrial vesicle formation, these observations further support our original conclusion that the vesicles are most likely to be autophagosomes. We also showed that the mitochondria forming autophagosomes stain positive for parkin. Quantification of parkin immuno-gold EM showed elevated levels of parkin in the cytosol and also increased parkin labeling on mitochondria-forming vesicles. These data imply that the mitochondrial vesicles represent a novel form of mitophagy. Moreover, inhibition of parkin by RNAi prevented an ICI (the antiestrogen known as Fulvestrant or Faslodex)-mediated reduction of mitochondrial content, supporting a role of parkin in mitochondrial clearance [1].

**Figure 2:**
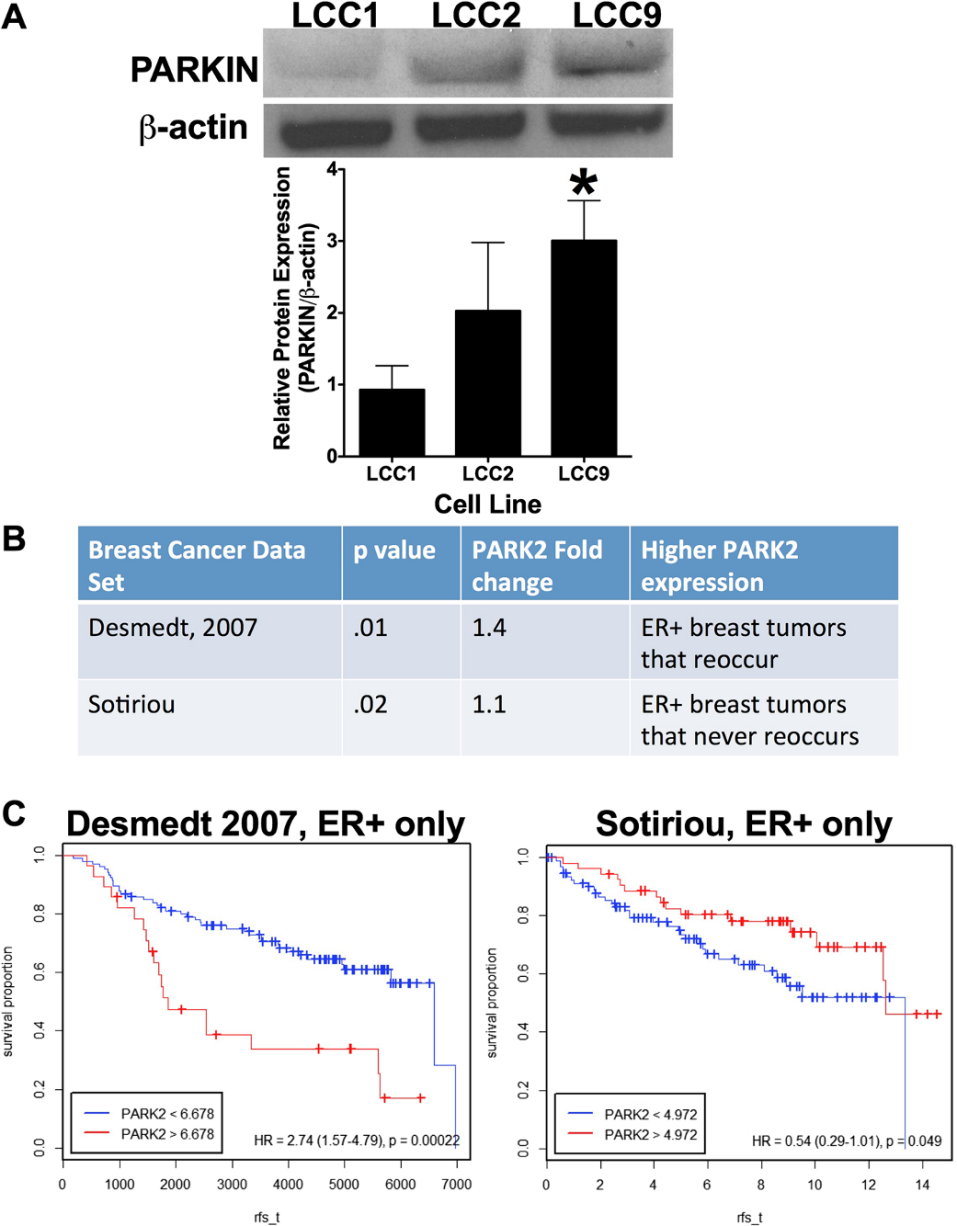
**Parkin expression in ER** + **breast cancer. A**. Parkin protein levels in ER + breast cancer cell lines as determined by Western blot hybridization. **B**. Parkin expression in ER + breast tumors that reoccur versus tumors that never reoccur. **C**. Parkin expression and ER + breast cancer survival in human data sets [10, 11].

Ding and Eskelinen discuss an interesting question on the role of parkin as a tumor suppressor. While we agree that in some cancers parkin may be a tumor suppressor [5], we find elevated endogenous levels of parkin in antiestrogen resistant LCC9 breast cancer cell lines when compared with their endocrine sensitive parental control cells (LCC1; Figure 2). Parkin was also shown to promote various cytoprotective cell signaling pathways including stabilization of the pro-survival BCL2 family member, MCL-1 [6]. BCL2 signaling is critically important to the maintenance of the antiestrogen resistance phenotype in ER + breast cancer cells [7–9], highlighting a possible pro-tumorigenic role of parkin in breast cancer. However, publically available human ER + breast cancer data sets are inconclusive on the role of parkin in ER + breast cancer survival (Figure 2B and 2C), where the results differ depending on the data sets analyzed [10, 11]. We also showed in our original manuscript that knockdown of PINK1 restores antiestrogen sensitivity to LCC9 breast cancer cells, further indicating a possible role of mitophagy in maintaining an endocrine therapy resistant phenotype [1]. PINK1 is a mitochondrial serine/theorinine kinase involved in the recruitment of parkin to the mitochondrial membrane. While PINK1 is predominately a mitochondrial protein, multiple reports have identified a cytosolic version of PINK1 [12, 13]. As we do not know the precise role of cytosolic PINK1 versus mitochondrial PINK1 in ER + breast cancer, we cannot exclude the possibility of both cytosolic and mitochondrial PINK1 contributing to the antiestrogen resistance phenotype. Further experimentation is needed to determine the role of parkin, PINK1, and mitophagy in antiestrogen resistance and breast cancer survival, which was outside the scope of our original short report.

**Figure 3:**
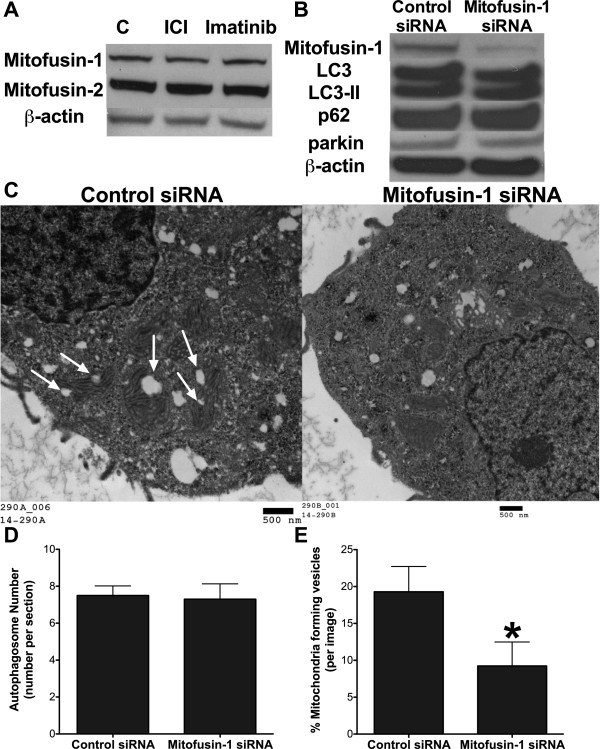
**Effect of mitofusin**-**1 on mitochondrial vesicle formation. A**. Mitofusin-1 and mitofusin-2 protein levels in LCC9 cells treated with 100 nM ICI or 10 µM Imatinib for 72 hours. **B**. Knockdown of mitofusin-1 in LCC9 cells was confirmed by Western blot hybridization and mitofusin-1 knockdown had no effect on either parkin expression or autophagosome formation. **C**. EM micrographs of LCC9 cells treated with control or mitofusin-1 siRNA. Arrows denotes mitochondrial forming vesicles. **D**. Autophagosomes were counted from EM images of LCC9 cells treated with control or mitofusin-1 siRNA. n = 10; *p > 0.05. **E**. Mitochondria forming vesicles were counted from EM images of LCC9 cells treated with control or mitofusin-1 siRNA. Data was graphed as % mitochondrial forming vesicles per image. n = 10; *p > 0.05.

Using a mouse embryonic fibroblast cell line, a previous study by Hailey *et al*. showed that mitochondria donate outer membrane material to form autophagosomes under serum starvation [14]. These authors showed that the mitochondrial membrane formation of autophagosomes is an ATG5 dependent and mitofusin-2 dependent process. Mitofusin-2 was critical to maintain the endoplasmic reticulum/mitochondrial connection necessary for mitochondrial autophagosome formation, and deletion of mitofusin-2 inhibited their formation [14]. The mitochondrial spheroid formation process illustrated in Ding *et al*., also using a mouse embryonic fibroblast cell line, indicated that the “mitochondrial spheroid” formation induced by the mitochondrial decoupling agent CCCP, is dependent on mitofusin-1 and mitofusin-2. The work by Ding *et al*. and Hailey *et al*. highlight the importance of mitofusin proteins in mitochondrial vesicle formation. While our original short report did not explore the effect of mitofusins, we now include data to begin to address their role. Mitofusin-1 and −2 levels do not change in LCC9 cells when treated with 100 nM ICI or 10 µM Imatinib for 72 hours (Figure 3A), the drugs utilized in Cook *et al*. to increase mitochondrial autophagosome formation. Knockdown of mitofusin-1 by RNAi had no effect on autophagosome formation as measured by LC3-II formation and p62 degradation (Figure 3B). Furthermore, inhibition of mitofusin-1 had no effect on parkin levels (Figure 3B), suggesting that there was no reciprocal relationship between parkin and mitofusins in human ER + breast cancer cells, unlike the report using mouse embryonic fibroblasts by Ding *et al*.

Consideration of cellular context is usually critical in the interpretation of much cell and molecular biologic data. Cellular signaling in cancer is often altered to favor proliferation and survival. While Ding and Eskelinen questioned our study due to differences they observed between parkin and mitofusin regulation, it would not be unusual for mitochondrial vesication to be controlled differently between a human ER + breast cancer cell line and a mouse embryonic fibroblast cell line.

The observations from western hybridizatons were confirmed in EM images taken from LCC9 cells transfected with mitofusin-1 siRNA (Figure 3C). Quantification of EM micrographs indicates that mitofusin-1 inhibition had no effect on autophagosome number (Figure 3D) when compared with control siRNA transfected cells. However, mitofusin-1 knockdown inhibited mitochondrial vesicle formation (Figure 3E). These data suggest that mitofusin-1 plays a critical role in the development of mitochondrial autophagosomes with no effect on the classical autophagosome formation pathways. These new data, coupled with our previous data, strongly support our original interpretation that, in ER + breast cancer cells, mitochondria donate their cellular membrane material to form autophagosomes and that this occurs in an ATG7 and mitofusin-1 dependent manner.

Finally, we appreciate that this is a controversial area and that others may choose to arrive at different conclusions from the same data. We appreciate the opportunity the journal has provided to contrast our interpretations with those of Ding and Eskelinen. We also look forward to the publication of additional studies that may better delineate the nature and physiological relevance of both autophagosomes and what appear to be the closely related “mitochondrial spheroid” structures.
